# Functional Profiling of Live Melanoma Samples Using a Novel Automated Platform

**DOI:** 10.1371/journal.pone.0052760

**Published:** 2012-12-28

**Authors:** Adam Schayowitz, Greg Bertenshaw, Emiko Jeffries, Timothy Schatz, James Cotton, Jessie Villanueva, Meenhard Herlyn, Clemens Krepler, Adina Vultur, Wei Xu, Gordon H. Yu, Lynn Schuchter, Douglas P. Clark

**Affiliations:** 1 BioMarker Strategies, Baltimore, Maryland, United States of America; 2 Melanoma Research Center, The Wistar Institute, Philadelphia, Pennsylvania, United States of America; 3 Abramson Cancer Center, University of Pennsylvania School of Medicine, Philadelphia, Pennsylvania, United States of America; 4 Department of Pathology and Laboratory Medicine, University of Pennsylvania School of Medicine, Philadelphia, Pennsylvania, United States of America; University of Colorado, United States of America

## Abstract

**Aims:**

This proof-of-concept study was designed to determine if functional, pharmacodynamic profiles relevant to targeted therapy could be derived from live human melanoma samples using a novel automated platform.

**Methods:**

A series of 13 melanoma cell lines was briefly exposed to a BRAF inhibitor (PLX-4720) on a platform employing automated fluidics for sample processing. Levels of the phosphoprotein p-ERK in the mitogen-activated protein kinase (MAPK) pathway from treated and untreated sample aliquots were determined using a bead-based immunoassay. Comparison of these levels provided a determination of the pharmacodynamic effect of the drug on the MAPK pathway. A similar ex vivo analysis was performed on fine needle aspiration (FNA) biopsy samples from four murine xenograft models of metastatic melanoma, as well as 12 FNA samples from patients with metastatic melanoma.

**Results:**

Melanoma cell lines with known sensitivity to BRAF inhibitors displayed marked suppression of the MAPK pathway in this system, while most BRAF inhibitor-resistant cell lines showed intact MAPK pathway activity despite exposure to a BRAF inhibitor (PLX-4720). FNA samples from melanoma xenografts showed comparable ex vivo MAPK activity as their respective cell lines in this system. FNA samples from patients with metastatic melanoma successfully yielded three categories of functional profiles including: MAPK pathway suppression; MAPK pathway reactivation; MAPK pathway stimulation. These profiles correlated with the anticipated MAPK activity, based on the known BRAF mutation status, as well as observed clinical responses to BRAF inhibitor therapy.

**Conclusion:**

Pharmacodynamic information regarding the ex vivo effect of BRAF inhibitors on the MAPK pathway in live human melanoma samples can be reproducibly determined using a novel automated platform. Such information may be useful in preclinical and clinical drug development, as well as predicting response to targeted therapy in individual patients.

## Introduction

Molecularly targeted agents (MTAs) that block specific, critical signal transduction pathways in malignant cells have emerged as major tools in the treatment of cancer. To be most effective, these drugs must be paired with a predictive test to match the right agent with the signaling defect harbored by the patient’s tumor. However, despite some successes, most cancer patients do not yet benefit from such personalized cancer care [1]–[2]. Part of the reason for this unmet need is due to the fact that the currently available predictive tests do not provide any direct information regarding signal transduction in patients’ cancer cells [3]. While the actual targets of most molecularly targeted agents are elements of the signal transduction network, most predictive tests only provide indirect and inferential information about signal transduction. These tests typically consist of DNA analysis for mutations in genes encoding activated signal transduction proteins or immunohistochemistry of over-expressed receptor tyrosine kinase proteins in fixed tissue. In reality, the goal of MTAs is not only to target individual proteins, but also to control the dynamic, complex circuitry of signal transduction that leads to tumor cell survival and proliferation. Such networks are not simple, linear pathways, but rather involve complex bypass mechanisms and feedback loops that are impossible to assess using genetic analysis [4]. This complexity often undermines successful MTA development and therapy [5]–[7].

MTA-related signaling complexities have been highlighted by the recent challenges posed by the development of BRAF inhibitors and other MAPK pathway inhibitors for metastatic melanoma [8]. It is clear that the subset of melanoma patients with activating mutations in BRAF derive the most benefit from MAPK pathway inhibitors such as vemurafenib, dabrafenib, and trametinib, but individual patient responses are variable and, more importantly, transient due to the rapid development of resistance. Interestingly, this resistance is not due to the emergence of secondary mutations in the kinase domain of BRAF, but rather through a variety of other mechanisms that reactivate the MAPK pathway and/or through the activation of bypass networks such as those involving the PI3K/AKT pathway [9]–[14]. Such signal transduction-mediated resistance is not limited to melanoma since it was recently found that BRAF-mutant colorectal carcinomas might subvert BRAF inhibitor monotherapy through a feedback loop involving activation of EGFR [15]–[16]. Fortunately, many approved and emerging drugs already exist to block these feedback loops and bypass mechanisms, provided that adequate tests are available to guide the selection of effective combination therapies.

To that end, we have created an automated, robust, reproducible, and potentially widely disseminated system for processing unfixed, fresh tumor samples from individual patients. This system enables “ex vivo” (that which takes place outside an organism) modulation and subsequent analysis of dynamic signal transduction networks in tumor biopsy samples to generate what we term a functional signaling profile [17]. In this proof-of-concept study we have focused on the ex vivo modulation of the MAPK pathway in melanoma cells by a BRAF inhibitor; however, the analysis of other relevant pathways is also possible. We have successfully generated functional signaling profiles from fine needle aspiration (FNA) biopsies of preclinical murine xenograft models of melanoma, as well as FNA biopsies of human metastatic melanoma. This system provides the unique opportunity to assess the pharmacodynamic effects of MTAs on individual patient samples during drug development, and may serve as the foundation for predictive tests to guide targeted therapy.

## Methods

### Cell Culture and Growth Inhibition Assays

Melanoma cell lines (A2058, COLO 829, Malme-3M, RPMI-7951, SK-MEL-2, SK-MEL-3, SK-MEL-28 and SK-MEL-31) were purchased from the American Type Culture Collection (Manassas, VA) and were maintained per ATCC instructions. The establishment and maintenance of the 451Lu, 451Lu-R, Mel1617, Mel1617-R, WM983B and WM983B-R cell lines has been described previously [9]. For growth inhibition assays, all cell lines were routinely cultured in the standard growth media described above. Cell proliferation assays were performed using the MTT assay (Sigma), to examine the effect of exposure to increasing concentrations of PLX-4720, a BRAF inhibitor that is structurally similar to the approved BRAF inhibitor, vemurafenib (Selleck Chemicals, Houston TX). The results were expressed as a percentage of the cell number in control wells. The IC50 values for PLX-4720 were calculated using non-linear regression (sigmoidal dose response) of the plot of percentage inhibition versus the log of inhibitor concentration in GraphPad Prism (v5; GraphPad Software, Inc., La Jolla, CA). Biological triplicates from three individual experiments were treated for _72_ hours with 3 nM to 30 uM PLX-4720. Error bars represent SEM.

### Instrumentation and Sample Processing

Cell lines, xenografts and human samples were processed on SnapPath™ (BioMarker Strategies, Baltimore MD), an automated cell and tissue processing platform which evokes functional signaling profiles from live cell and biopsy samples. Briefly, fresh cell and biopsy samples, containing a mixture of live and dead cells, were loaded onto the instrument in 500 ul of supportive media. Samples were then moved through five distinct processing steps on the instrument utilizing automated fluidics. Samples were first dispersed into a more homogeneous cellular suspension using mechanical shear forces. Next, samples were enriched for tumor cells by immunodepleting the samples of CD45-positive non-tumor cells (lymphocytes and macrophages) with a short 2 minute incubation with anti-CD45 antibodies conjugated to magnetic Dynal beads (Life Technologies, Carlsbad, CA) then separating out bead-bound cells by a magnet on the platform. The sample was then aliquoted into two equally-sized separate temperature-controlled test chambers and treated with either vehicle (DMSO) or PLX-4720 (3 uM final concentration) and incubated at 37 C for 5 min. Samples were then lysed and stabilized using a cell lysis buffer (20 mM Tris-HCl, pH 7.5, 150 mM NaCl, 1% Triton, 1 mM Na_2_EDTA, 1 mM EGTA, 2.5 mM sodium pyrophosphate, 1 mM beta-glycerophosphate, 1 mM Na_3_VO4, 1 µg/ml leupeptin and 1 mM PMSF; Cell Signaling, Boston, MA). Replicates were generated by additional runs on the instrument. For cell lines the number of replicates ranged from 4 to 8. For xenografts, the number of replicates was 4. For clinical human samples, which unlike cell lines and xenografts cannot be replenished, each was processed on the instrument once. Once generated, lysates were analyzed off the instrument.

### Bio-Plex Immunoassays

For each sample, p-ERK was measured in cell lysates from both the control and PLX-4720 test chamber aliquots on the Bio-Plex™ 200 System using a bead-based immunoassay that detects both p-ERK1 (Thr202/Tyr204) and p-ERK2 (Thr185/Tyr187)(Bio-Rad Laboratories, Hercules, CA). Each sample was analyzed in triplicate and results mean averaged. Data were acquired using Bio-Plex Manager™ Instrument Control (v6), then extracted and analyzed using GraphPad Prism (v5; GraphPad Software, Inc., La Jolla, CA). Levels of p-ERK detected in the control test chamber were compared with those in the PLX-4720 chamber and a percent inhibition level was determined based on the difference. For Bio-Plex analysis, inputs were normalized by total protein concentration using the Pierce® 660 nm Protein Assay (Thermo Scientific, Rockford, IL, USA). For cell lines and xenografts, the lysate concentration was 200 ug/ml and 140 ug/ml, respectively. For clinical samples, the lysates were analyzed at the highest possible concentration with a range of 25–300 ug/ml. For a given control-PLX-4720 sample pair, the protein concentration was equalized using lysis buffer.

### Western Blot Immunoassays

For SK-MEL-28, SK-MEL-2, A2058 and RPMI-7951 cell lines, we measured p-ERK1 (Thr202/Tyr204) and p-ERK2 (Thr185/Tyr187) using rabbit anti-p-ERK1/2 (Cell Signaling; Clone D13.14.4E; 1∶500 dilution) and goat anti-rabbit IgG conjugated with horseradish peroxidase (Bio-Rad; 1∶3000). Total ERK1/2 was measured using rabbit anti-ERK1/2 (Cell Signaling; Clone 137F5; 1∶1000) and goat anti-rabbit IgG conjugated with horseradish peroxidase. As a loading control, we measured actin using mouse anti-beta-actin (Sigma, St Louis, MO;, A2228; 1∶2000) and goat anti-mouse IgG conjugated with horseradish peroxidase (Bio-Rad: 1∶3000). For, p-ERK and total ERK, 10 ug of each lysate was subjected to reducing and denaturing PAGE using precast 10% PAGE Tris-HCl gels (Bio-Rad). For beta-actin, 5 ug of each lysate was used. Proteins were then transferred to nitrocellulose for probing. The Pierce ECL Western Blotting Substrate (Thermo) was used as recommended by the manufacturer. Film was scanned and densitometry conducted using ImageJ (NIH, Bethesda, MD).

### Xenografts

Athymic nu/nu female mice 4–6 weeks of age were obtained from Harlan Laboratories and were housed in a pathogen-free environment under controlled conditions of light and humidity and received food and water ad libitum. The mice were inoculated with SK-MEL-28, MALME-3M, COLO829 or A2058 cell lines obtained from ATCC. The cells were grown in regular growth media as described above. Subconfluent cells were removed from culture, then resuspended in Matrigel at 2×10^7^ cells/ml. Each mouse received a subcutaneous inoculation at one site per flank with 100 ul of cell suspension. Tumor formation varied between 4 and 8 weeks by cell line. Upon reaching 500 mm^3^ FNA biopsies were performed. One FNA sample consisted of 4 passes of a 23G, 1” needle on a 10cc syringe. Samples were then processed on the SnapPath as described above. Following biopsy, all mice were euthanized by CO_2_ and cervical dislocation.

### Human Subjects

Subjects were enrolled who had a known history of metastatic melanoma and either subcutaneous metastases amenable to palpation-guided fine needle aspiration biopsy or who were undergoing surgical resection of a metastatic lesion for clinical purposes. The clinical status of each patient at the time of biopsy, including current therapy and current response to therapy, was determined by the oncologist based on clinical assessment and available medical records. Tumor BRAF status was obtained from the individual patients medical record based on clinical testing.

For the subcutaneous lesions, samples were collected using standard fine needle aspiration biopsy techniques by either an experienced cytopathologist or an oncologist who was trained and experienced in the procedure. For the surgically-excised lesions, specimens were delivered within 30 min. of excision to the pathology department where a cytopathologist examined the specimen and performed the FNA procedure on the specimen. FNA Samples were delivered within 1 hour to the laboratory for ex vivo processing on the on SnapPath™ platform.

### Ethics Statement

This study was carried out in strict accordance with the recommendations in the Guide for the Care and Use of Laboratory Animals of the National Institutes of Health. The protocol was approved by the Committee on the Ethics of Animal Experiments of Sobran, Inc., Baltimore, MD (Protocol Number: BIO-001-2010). The human subjects protocol and consent form were approved by the Institutional Review Boards (IRBs) of the University of Pennsylvania and Abramson Cancer Center’s Clinical Trials Scientific Review and Monitoring Committee. The study was conducted in compliance with regulations of the Health Insurance Portability and Accountability Act and the Declaration of Helsinki. Informed written consent was obtained for the biopsy procedure prior to enrollment.

## Results

### Automated Platform (SnapPath™) for ex vivo Generation of Functional Signaling Profiles

Our goal was to develop a reproducible and potentially distributable system to profile dynamic signal transduction events in living tumor cells from individual patients. During the development process our priorities included: the ability to accommodate multiple sample types; the inclusion of small samples from minimally invasive biopsies like FNAs; the ability to provide relatively rapid results; and automation to provide robust and reproducible results.

**Figure 1:**
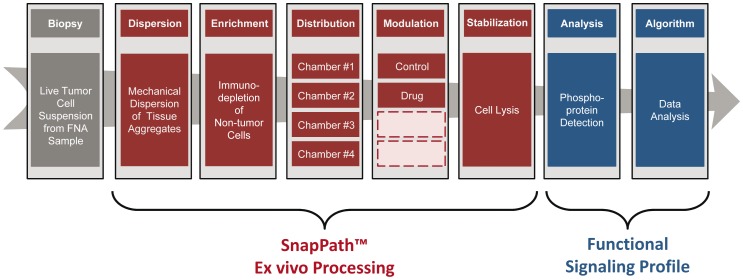
Overview of the automated system (SnapPath™) to generate functional signaling profiles (FSP) from live tumor samples. This system employs the SnapPath instrument, an automated fluidics device, to move a suspension of live tumor cells through various steps to evoke measurable changes in signal transduction ex vivo. These five sequential steps include: (1) dispersion of the tissue fragments into a homogeneous suspension using mechanical shear forces; (2) enrichment of the sample for tumor cells by immunodepleting non-tumor cells; (3) distribution of equal aliquots into multiple test chambers; (4) modulation of signal transduction pathways by exposure to drugs or growth factors; and (5) stabilization of the sample through cell lysis. Once processed, samples can be analyzed off-platform. In the current use, we used a bead-based immmunoassay to analyze the phosphoprotein p-ERK1/2 levels in output samples. Finally, an algorithmic analysis of data can be used to generate a functional signaling profile of the tumor. In the current use, we used a simple cut-off value of percent inhibition of p-ERK1/2.

In the resultant system, suspensions of live tumor cells are placed onto an automated fluidics-based platform where the samples are dispersed using mechanical shear forces into a more homogeneous suspension of individual cells and small cell clusters (Figure 1). The samples are then enriched for tumor cells by immunodepleting the samples of non-tumor cell types such as lymphocytes, using antibody-coated magnetic beads. The actual FNA procurement process enriches for tumor cells, thus samples contain very few fibroblasts or endothelial cells. The most common non-tumor cell contaminants are lymphocytes. Therefore, we developed the capability of the platform to remove these cells. The instrument has the ability to remove >80% of CD45+ cells. The tumor cell-enriched samples are then aliquoted into multiple individual test chambers containing reagents to modulate signal transduction in the tumor cells, such as drugs or growth factors. After a brief incubation (from 5 min. to 4 hours) with the modulating agent, the samples are lysed for subsequent analysis of phosphoproteins within signal transduction pathways of interest. With a 5 min. modulation step, this entire process requires approximately 30 min. In order to generate data on numerous phosphoproteins from small samples, we utilize a sensitive bead-based immunoassay capable of multiplexing (Bio-Plex). This off-instrument assay has allowed us to successfully assay as few as 50,000 cells. The functional signaling profile is then derived from a comparison of phosphoprotein biomarker levels in the control, unmodulated test chamber to those in the modulated test chambers. For example, in these studies we compared the levels of p-ERK in control aliquots to those treated with the BRAF inhibitor PLX-4720.

### Growth Inhibition of Melanoma Cell Lines by a BRAF Inhibitor (PLX-4720)

**Figure 2:**
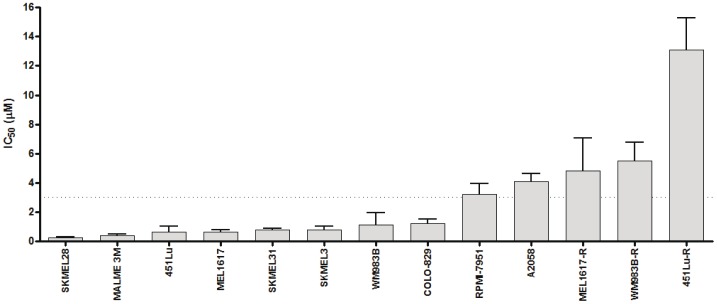
IC50 Values of a panel of melanoma cell lines exposed to the BRAF inhibitor, PLX-4720. The relative sensitivity of a panel of melanoma cells lines containing V600E BRAF mutations was determined using the MTT assay to assess cell proliferation in the presence of PLX-4720. Five cell lines were determined to be resistant (IC50>3 uM). Three of these resistant lines (MEL1617-R, WM983B-R, 451Lu-R) were previously generated from respective sensitive parental cell lines (MEL1617, WM983B, 451Lu) by exposure to increasing concentrations of a BRAF inhibitor.

As a foundation for subsequent studies in murine xenografts and humans, a series of 13 BRAF V600E mutant melanoma cell lines was tested for sensitivity to BRAF inhibition by determining the IC50 values of each cell line to PLX-4720. As anticipated, 8 of the cell lines showed marked sensitivity to PLX-4720 (IC50<3 uM) (Figure 2) [18]–[19]. Despite containing a BRAF V600E mutation, five cell lines were resistant to PLX-4720 (IC50>3 uM). Included in these resistant lines were three previously generated cell lines (MEL1617-R; WM983B-R; 451Lu-R) that had been selected for resistance by exposure to increasing concentrations of a BRAF inhibitor (SB-590885) [9].

### Functional Signaling Profiles Uniquely Stratify Melanoma Cell Lines

Next, functional signaling profiles were generated from each of the cell lines using the previously described automated platform. Briefly, tumor cells were grown to ∼70% confluence on 10 cm dishes, and then suspensions of tumor cell lines (1–3 million cells) that had been removed from culture plates were processed on the automated platform, which included exposure to PLX-4720 for 5 min., then levels of p-ERK were assessed from aliquots that had been treated or untreated with PLX-4720 using an immunoassay. For each sample, the level of the p-ERK phosphoprotein in the untreated aliquot was compared to the level in the PLX-4720-treated aliquot and a percent inhibition was generated such that 100% inhibition represented complete suppression of the MAPK pathway marker, p-ERK, and 0% inhibition represented complete inactivity of PLX-4720 on the MAPK pathway.

**Figure 3:**
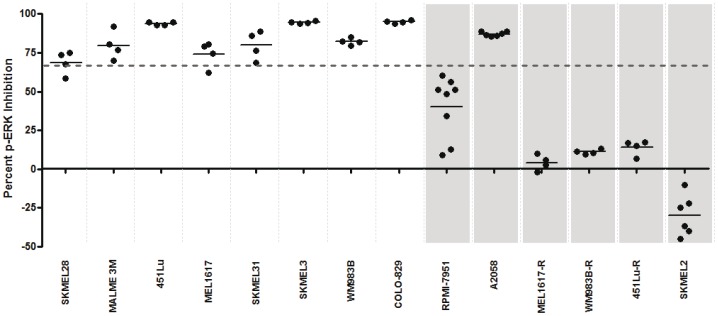
Functional signaling profiles of a panel of melanoma cell lines exposed to the BRAF inhibitor, PLX-4720. A panel of fourteen melanoma cell lines was exposed to PLX-4720 using the automated platform. Cell lines previously determined to be resistant to BRAF inhibition are indicated by grey shaded columns. All cell lines contain a BRAF V600E mutation except SKMEL2, which is wild type for BRAF but contains an NRAS mutation. The percent inhibition of p-ERK levels relative to a control untreated aliquot is indicated for each sample, with each dot representing an individual replicate. A dotted line at 66% inhibition indicates a cut value that stratifies most BRAF inhibitor sensitive cell lines from resistant lines. In the SKMEL2 sample, p-ERK levels were increased by exposure to PLX-4720, relative to the untreated aliquot.

**Figure 4:**
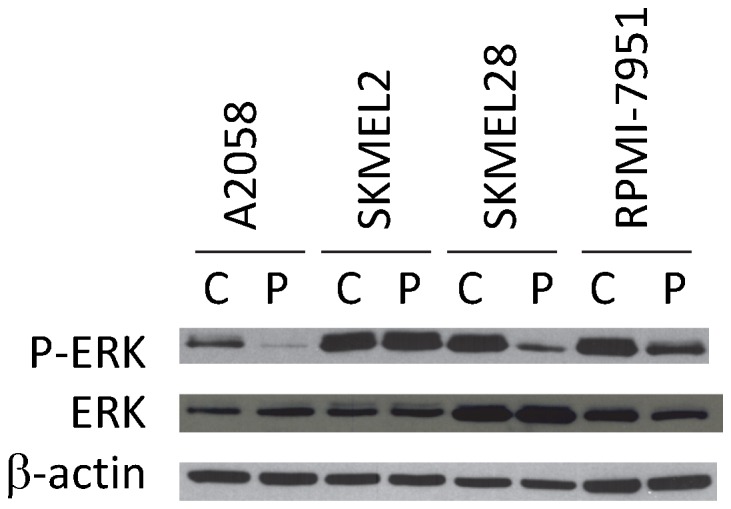
Western Blot analysis of melanoma cell lines exposed to the BRAF inhibitor, PLX-4720. A panel of four melanoma cell lines was exposed to PLX-4720 using the automated platform and then lysates were analyzed by Western Blot analysis. For each lysate, p-ERK, total ERK and beta-actin levels were determined. Percent inhibition of p-ERK levels was determined using densitometry by determining the ratio of the p-ERK signal for a vehicle control sample (C) compared to the PLX-4720-treated sample (P).

In this analysis, 9 of the 14 cell lines displayed substantial ex vivo PLX-4720 suppression of the MAPK pathway (69–95% mean p-ERK inhibition) (Figure 3). Eight of these nine lines displayed sensitivity to PLX-4720 in our previous IC50 assessment. Interestingly, the intrinsically BRAF inhibitor-resistant line, A2058, displayed functional evidence of MAPK pathway blockade by PLX-4720 (88% mean inhibition), suggesting an alternate resistance mechanism. Four of the remaining lines showed intact activity of the MAPK pathway (4–30% mean p-ERK inhibition) despite exposure to PLX-4720 (Figure 3). Each of these cell lines was determined to be a PLX-4720-resistant line based on our previous analysis (Figure 2). Finally, a functional profile was also obtained from a cell line (SKMEL2) with a wild type BRAF gene and a known NRAS mutation (Q61R) that displays a high level of resistance to PLX-4720 [18]. Interestingly, in this cell line, exposure to PLX-4720 increased the activity of the MAPK pathway (30% mean stimulation). This paradoxical MAPK pathway up-regulation by BRAF inhibitors in NRAS mutant cells has been previously described [20]–[23], but reinforces the validity of our approach in monitoring MAPK pathway dynamics. Based on all of these cell line data, we visually established an arbitrary cut value of 66% inhibition to distinguish BRAF inhibitor sensitive lines from resistant lines using our functional profiling assay. This cut value is also consistent with the previous evidence that BRAF inhibitors must have a significant impact on the MAPK pathway in order to be clinically effective [24]. In addition, the paradoxical up-regulation of the MAPK pathway in the setting of NRAS mutations was also detected in this functional assay, creating three distinct categories of functional signaling profiles. To verify the Bio-Plex assay data, we have conducted Western blot analysis. We utilized 4 cell lines that are representative of all lines used in the study. Namely, the SK-MEL-28 cell line that is sensitive to PLX-4720 in vivo and ex vivo, SK-MEL-2 that is resistant to PLX-4720 in vivo and ex vivo, A2058 that is resistant to PLX-4720 in vivo but sensitive ex vivo and finally RPMI-7951 that has intermediate sensitivity to PLX-4720 in vivo and ex vivo. For all four cell lines, the Western blot data corroborated the Bio-Plex data (Figures 3 and 4). For A2058 cells, the p-ERK inhibition was 92.1% by Western, based on densitometry, and 87.0% by Bio-Plex. For SK-MEL-28, RPMI-7951 and SK-MEL-2 the inhibition was 73.8% and 68.8%, 37.4% and 40.4%, and −10% and −29.8%, respectively.

### Functional Signaling Profiling of Melanoma Xenograft FNA Samples

The ultimate value of this system lies in its ability to generate functional information from relatively small, clinically relevant samples. In addition, it has the potential to streamline preclinical studies utilizing human tumor cell-based xenografts or murine tumorgrafts of patient derived samples. To that end, we have optimized protocols to generate functional profiles from fine needle aspiration biopsy (FNA) samples. Murine models of metastatic melanoma resemble human subcutaneous melanoma metastases, displaying similar biology, histopathology, and FNA cytopathologic features [25]. Four of the previously characterized cell lines were propagated as xenografted tumors in mice, including three PLX-4720-sensitive lines (SKMEL28, MALME3M; COLO829) and one PLX-4720-resistant line (A2058). FNA biopsies were performed on each model.

**Figure 5:**
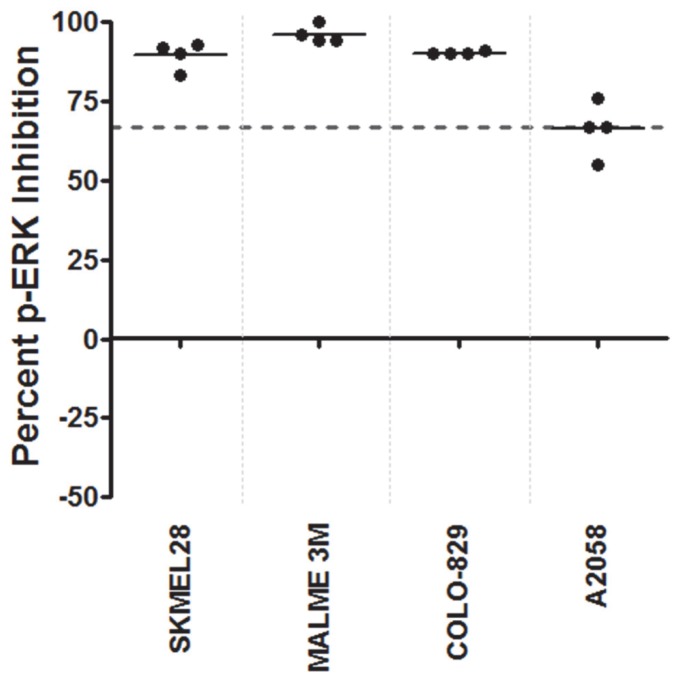
Functional Signaling profiles of melanoma xenograft FNA samples exposed to the BRAF inhibitor, PLX-4720. FNA samples were procured from a set of four different melanoma xenograft tumor models (SKMEL28, MALME3M, COLO829, A2058) and ex vivo functional profiles were obtained using the automated platform. The percent inhibition of p-ERK levels relative to a control untreated aliquot is indicated for each sample, with each dot representing an individual FNA sample from a unique tumor. A dotted line at 66% inhibition indicates a cut value that stratifies most BRAF inhibitor sensitive cell lines from resistant lines.

Ex vivo functional profiling was then performed on the FNA-procured tumor cell suspensions from each xenograft model, using the automated platform, as previously described for the cell lines. Despite variable cell counts (0.60 to 19×10^6^) and tumor cell viability (14.6–51.0%) between FNA biopsy samples, successful functional profiles were obtained from most samples. The mean percent inhibition of these samples following PLX-4720 treatment ranged from 66–96% (Figure 5). Within each xenograft model, the inter-sample and inter-tumor variability was low, with standard deviations of p-ERK inhibition of 0.5% in COLO-829 to 8.62% in A2058. The mean percent p-ERK inhibition was similar between the previous cell line analysis (Figure 3) and the corresponding xenograft FNA samples (SKMEL28∶69 vs. 89%; MALME3M: 80 vs. 96%; COLO829∶95 vs. 90%; A2058∶88 vs. 66%)(Figure3 and 5). While some differences exist between the cell lines and the xenograft data, this may be due to different biology between the 2-dimensional cultures and the xenografted cell line tumors. Despite these differences, the cell lines and respective tumors still fall within the same functional category of MAPK activity. These studies indicate that functional profiling can be performed on preclinical xenograft models of melanoma and suggest that similar studies could be performed on human samples.

### Functional Signaling Profiles Generated from Human Metastatic Melanoma Biopsy Samples

**Figure 6:**
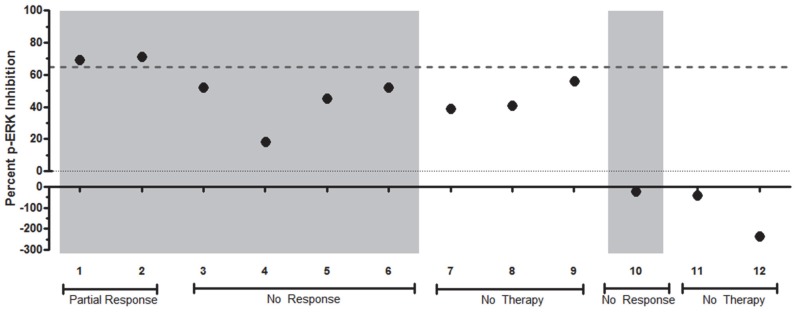
Functional Signaling profiles of human melanoma patient samples. Twelve human melanoma samples derived from FNA samples of subcutaneous lesions (#3, 4, 5, 7, 8, 10) or FNA samples of surgically-excised lesions (#1, 2, 6, 9, 11, 12) were exposed to PLX-4720 ex vivo using the automated platform. The percent inhibition of p-ERK levels relative to a control untreated aliquot is indicated for each sample. A dotted line at 66% inhibition was previously found to stratify BRAF inhibitor sensitive cell lines from resistant lines. The grey shaded columns indicate tumors that are known to be BRAF mutant (all V600E, except #6 which was V600K); un-shaded columns are BRAF wild type. Two samples showed a substantial decrease of p-ERK levels by PLX-4720 (#1 and 2), consistent with MAPK pathway suppression. Seven samples (#3–9) showed sustained p-ERK levels in the presence of PLX-4720, consistent with persistent MAPK activity. In three samples (#10, 11 and 12) the p-ERK levels were paradoxically increased by exposure to PLX-4720. The clinical response to BRAF inhibitor therapy at the time of biopsy is noted; patients with no history of BRAF inhibitor therapy are also indicated.

Functional profiling was then performed on a set of human samples derived from patients with metastatic melanoma. These samples were procured by FNA, either directly from subcutaneous metastatic lesions (n = 6) or from surgically-excised metastases (n = 6). Cell numbers in the samples ranged from 0.228–5.95×10^6^ for subcutaneous metastases and 5.70–183×10^6^ for surgically-excised samples. As in the previous preclinical studies, the functional profiling of these samples involved brief exposure to PLX-4720 on the automated platform followed by stabilization and subsequent analysis of p-ERK in untreated and treated aliquots of the sample to assess the ex vivo effect of the drug on the MAPK pathway. Of the twelve samples, two (#1 and 2) displayed substantial ex vivo PLX-4720 suppression of the MAPK pathway (69 and 71% inhibition), seven (#3–9) showed intact MAPK pathway activity (18–56% inhibition), and three (#10–12) showed MAPK pathway activation (21–235% stimulation) upon ex vivo PLX-4720 exposure (Figure 6). Two samples (#8 and 9) were from the same patient and the same subcutaneous flank lesion, procured several days apart via FNA and the surgically-excised specimen, respectively. These samples showed similar levels of MAPK inhibition (41 and 56%).

Correlation of these functional profiles with the known tumor BRAF genotype and clinical response to BRAF inhibitors revealed that the two patients with substantial ex vivo PLX-4720 suppression of the MAPK pathway (#1 and 2) were the only two patients whose tumors contained the BRAF V600E genotype and who showed at least a partial response to BRAF inhibitor therapy. Of the seven samples with intact MAPK pathway activity, three were from patients that were wild type for BRAF (#7–9), which would be anticipated based on the known intrinsic resistance of BRAF wild type tumors to BRAF inhibition [18]. Interestingly, the remaining four samples that showed intact activity of the MAPK pathway despite ex vivo exposure to PLX-4720 (#3–6) were BRAF V600E/K patients who were not responding to BRAF or BRAF plus MEK inhibitors at the time of the biopsy. Of the three patients that showed MAPK pathway activation, two were wild type for BRAF (#11–12), and a third (#10) had the V600E mutation and was not responding to BRAF inhibitor therapy. In these three patients, the MAPK pathway activation suggests that these patients may have signal transduction network alterations that lead to paradoxical MAPK activation upon BRAF inhibition. Taken together, these results indicate that functional profiles can be generated from FNA biopsy samples from patients or surgically-excised tumors. In addition, these functional profiles generate novel information about individual patient tumor signal transduction circuitry that correlates with known genetics, biology and individual patient responses to drug therapy.

## Discussion

In this study we have described a novel, automated system for the ex vivo pharmacodynamic analysis of human melanoma samples. We have termed the results of such analyses functional signaling profiles. Our preliminary studies with melanoma cell lines confirmed that functional signaling profiles from this ex vivo system accurately predicted BRAF inhibitor sensitivity in virtually all cell lines, based on their genotype, known biology, and IC50 values. These studies were extended to a clinically-relevant model system, using fine needle aspiration biopsy samples of human melanoma xenografts, which resemble human FNAs of metastatic melanoma in their cell composition and procurement method [26]. These xenograft studies confirmed that functional signaling profiles can be generated from such samples and that these profiles resemble those found in the preliminary cell line work. Finally, we generated functional signaling profiles from a set of samples derived from patients, including FNAs of subcutaneous melanoma metastases, as well as FNAs of surgically-excised metastatic melanoma lesions. These profiles were consistent with the known melanoma genotype, therapeutic regimen, and clinical status on that regimen.

Due to the prevalence of BRAF mutations in melanoma, the MAPK pathway has emerged as a prime target of molecularly targeted agents for this disease, including an approved inhibitor of BRAF (vemurafenib) and additional emerging inhibitors of BRAF and MEK [27]–[31]. Despite success in extending overall survival in patients with BRAF V600E mutations, a spectrum of responses, ranging from no response to complete response, occurs even in this highly selected population. In addition, virtually all patients develop resistance to BRAF therapy, often through re-activation of the MAPK pathway. This suggests that many additional factors are important in the modulation and pharmacodynamics of MAPK pathway activity in these patients.

In our cell line work, the functional signaling profiles of the MAPK pathway identified ex vivo pathway blockade in every sensitive line, identified the known paradoxical pathway activation in an NRAS mutant line, and identified MAPK pathway reactivation in the known BRAF inhibitor resistant lines, with one interesting exception. The A2058 cell line contains a V600E mutation, displays ex vivo MAPK inhibition by PLX-4720, but is intrinsically resistant to BRAF inhibitors. While the exact mechanism of this resistance, despite pathway inhibition, is still under investigation, one study has suggested that it could be due to bypass pathway activation via PTEN loss and RB loss [32]. These findings are clinically relevant, since they suggest that in such patients, combination therapy should include continued use of the BRAF inhibitor, with addition of a PI3K pathway inhibitor, for example. Conversely, patients with ex vivo MAPK reactivation in the presence of a BRAF inhibitor will likely require a different MAPK inhibitor, such as a MEK inhibitor, and possibly a PI3K pathway inhibitor as has been previously proposed [9] [33]–[34].

The functional signaling profiles generated in this study stratify human samples into three groups that display the following key features in the ex vivo presence of BRAF inhibition: MAPK suppression; MAPK reactivation; and MAPK stimulation. Because of the enrollment criteria and the practice patterns at the referral center where the study was conducted, most of the patients in the study were either BRAF wild type or BRAF V600E patients who were clinically progressing on a MAPK inhibitor regimen (BRAF or BRAF+MEK inhibitors). Most of these patients displayed a MAPK reactivation profile, confirming the previous evidence that MAPK reactivation plays a major role in BRAF inhibitor resistance. Interestingly, several patients displayed a MAPK activation profile, suggesting the acquisition of a previously described MAPK activation mechanism [11], or a novel pathway activation mechanism. Finally, in this cohort there were two patients who displayed a MAPK suppression profile. Interestingly, these were the only patients who possessed a BRAF mutation, were receiving a BRAF inhibitor, and showed clinical signs of response.

In summary, in this proof-of-concept study we have successfully generated pharmacodynamic data from human melanoma samples using a novel, automated ex vivo platform. This study focused on the MAPK pathway in metastatic melanoma, but this system is applicable to any solid tumor and multiple other molecularly targeted agents and signaling pathways. This system has potential applications in the efficient performance of preclinical drug studies in murine xenograft or human tumorgraft models. Also, it represents a novel tool for early clinical drug development in the assessment of pharmacodynamic responses to investigational drugs. Finally, it also has the potential to serve as a powerful predictive test to stratify patients in late stage clinical trials, and ultimately to guide targeted therapy.
